# Investigating Substance Use via Reddit: Systematic Scoping Review

**DOI:** 10.2196/48905

**Published:** 2023-10-25

**Authors:** Yu Chi, Huai-yu Chen

**Affiliations:** 1 School of Information Science University of Kentucky Lexington, KY United States; 2 Department of Communication University of Kentucky Lexington, KY United States

**Keywords:** substance use, systematic scoping review, Reddit, social media, drug use, tobacco use, alcohol use

## Abstract

**Background:**

Reddit’s (Reddit Inc) large user base, diverse communities, and anonymity make it a useful platform for substance use research. Despite a growing body of literature on substance use on Reddit, challenges and limitations must be carefully considered. However, no systematic scoping review has been conducted on the use of Reddit as a data source for substance use research.

**Objective:**

This review aims to investigate the use of Reddit for studying substance use by examining previous studies’ objectives, reasons, limitations, and methods for using Reddit. In addition, we discuss the implications and contributions of previous studies and identify gaps in the literature that require further attention.

**Methods:**

A total of 7 databases were searched using keyword combinations including *Reddit* and substance-related keywords in April 2022. The initial search resulted in 456 articles, and 227 articles remained after removing duplicates. All included studies were peer reviewed, empirical, available in full text, and pertinent to Reddit and substance use, and they were all written in English. After screening, 60 articles met the eligibility criteria for the review, with 57 articles identified from the initial database search and 3 from the ancestry search. A codebook was developed, and qualitative content analysis was performed to extract relevant evidence related to the research questions.

**Results:**

The use of Reddit for studying substance use has grown steadily since 2015, with a sharp increase in 2021. The primary objective was to identify tendencies and patterns in various types of substance use discussions (52/60, 87%). Reddit was also used to explore unique user experiences, propose methodologies, investigate user interactions, and develop interventions. A total of 9 reasons for using Reddit to study substance use were identified, such as the platform’s anonymity, its widespread popularity, and the explicit topics of subreddits. However, 7 limitations were noted, including the platform’s low representativeness of the general population with substance use and the lack of demographic information. Most studies use application programming interfaces for data collection and quantitative approaches for analysis, with few using qualitative approaches. Machine learning algorithms are commonly used for natural language processing tasks. The theoretical, methodological, and practical implications and contributions of the included articles are summarized and discussed. The most prevalent practical implications are investigating prevailing topics in Reddit discussions, providing recommendations for clinical practices and policies, and comparing Reddit discussions on substance use across various sources.

**Conclusions:**

This systematic scoping review provides an overview of Reddit’s use as a data source for substance use research. Although the limitations of Reddit data must be considered, analyzing them can be useful for understanding patterns and user experiences related to substance use. Our review also highlights gaps in the literature and suggests avenues for future research.

## Introduction

### Background

Substance use disorder (SUD) is a critical topic with substantial social impact, affecting individuals, families, and society at large. The recovery journey can be challenging and complex, requiring support from a strong network, including medical professionals, peers, and family members [1,2]. However, many people with SUD are reluctant to disclose their situations and seek support from in-person groups owing to physical distance, time, or the stigma associated with addiction [3]. Consequently, social media platforms are playing an increasingly important role in providing peer support, information, and resources to individuals with SUD.

Research has shown that social media platforms offer several benefits for studying sensitive topics, such as their round-the-clock availability, anonymity, and immediate and time-delayed responses [4-6]. The large-scale and diverse discussions on social media have enabled previous researchers to reveal the topics around substance use, classify and model users’ distinct behaviors, and predict the transitions into recovery or addiction relapse [7-10].

Reddit (Reddit Inc) is particularly well suited for substance-related research because of its large user base and diverse communities. With >57 million daily active users and 13 billion posts and comments as of 2021, Reddit offers researchers access to a broad population of users and rich discussions [11]. In addition, Reddit’s anonymous nature allows users to discuss sensitive topics such as SUD without the fear of stigma or judgment. As a result, an increasing number of studies have used Reddit to study the use of and recovery from various types of substances with different research objectives. Despite the potential benefits of using Reddit as a data source for substance use research, there are several challenges and limitations that researchers should consider. For example, Reddit data can be challenging to collect and analyze because of the platform’s constantly changing content and user behavior. Furthermore, ethical considerations such as user privacy and consent must be carefully considered when using social media data [12].

To the best of our knowledge, no systematic scoping review has been conducted on the use of Reddit as a data source to study substance use. Therefore, this review is essential as it will provide an updated landscaping overview of research on this topic. By synthesizing and summarizing the existing literature, this review will highlight the current state of research, identify gaps, and provide insights for future studies. Furthermore, this review will explore the reasons and limitations of using Reddit for substance use research, thus contributing to the ongoing discussion on the use of social media data in research. The findings of this review will be of interest to researchers, clinicians, policy makers, and social media users interested in the role of social media in addressing SUDs. For researchers, the insights of this study illuminate both the strengths and challenges of conducting Reddit-based studies, guiding future research design. Clinicians and policy makers can benefit by understanding the nature of substance use conversations on Reddit, informing evidence-based interventions and policies. In addition, social media users may gain awareness of the breadth of SUD-related discussions, empowering them to engage thoughtfully with content and connect with supportive communities for healthier outcomes.

### Objectives

The objective of this review was to provide a comprehensive overview of the research that uses Reddit as a major data source to study substance use. Specifically, this review aims to identify the objectives of previous studies, examine the reasons and limitations of using Reddit, evaluate the methods for collecting and analyzing Reddit data, and discuss the implications and contributions of the studies. We aim to answer the following research questions (RQs):

- RQ1: What are the primary objectives of previous studies that used Reddit to study substance use?
- RQ2: What are the reasons for and limitations of using Reddit to study substance use, as reported by previous studies?
- RQ3: What methodological approaches have been used? How have previous studies collected and analyzed Reddit data to study substance use?
- RQ4: What are the main implications and contributions of previous studies that have used Reddit to study substance use?

## Methods

This systematic scoping literature review followed a 4-step process, which included literature search, data screening, data extraction, and synthesis, as outlined by Arksey and O’Malley [13]. We also adhered to the guidelines of PRISMA-ScR (Preferred Reporting Items for Systematic Reviews and Meta-Analyses extension for Scoping Reviews) [14] and the guidelines from the Joanna Briggs Institute Scoping Review Methodology Group [15,16].

### Literature Search

A total of 7 databases were identified for the literature search: PubMed, Web of Science, PsycINFO, Embase, ProQuest, Annual Reviews, and ACM Digital Library. Then, we combined the keyword *Reddit* with substance-related keywords for drug use (eg, substance, drug, opioid, opiate, marijuana, and cannabis), alcohol use (eg, alcohol and drinking), and tobacco use (eg, tobacco and smoking). The keyword search queries were carried out in the title, abstract, and topic fields, depending on the database (refer to Table S1 in Multimedia Appendix 1 [10,17-75] for search keywords and search dates by each database). The search was conducted from April 22, 2022, to April 24, 2022.

### Screening Procedure and Eligibility Criteria

The initial search in the 7 databases resulted in 456 articles, and 227 articles remained after removing duplicates. The first round of screening was performed on the titles and abstracts by YC and HC independently, and 35.7% (81/227) of the articles remained for full-text analysis. During the data extraction and synthesis stages, further screening was conducted to validate the eligibility of the articles by examining their content. Articles were excluded from our data set if they met any of the following exclusion criteria: (1) full text not available (eg, conference abstract), (2) irrelevant to Reddit, (3) irrelevant to substance use, (4) not an empirical study (eg, literature review), (5) not a peer-reviewed article, and (6) not in English.

### Data Extraction and Synthesis

We used a Google spreadsheet to extract and record the basic information of the included articles (eg, author, title, year of publication, and type of publication) and evidence relevant to our RQs (eg, study objective, reasons, and limitations of using Reddit). To develop our initial codebook, we conducted an open coding process on 12 randomly selected articles, which was subsequently refined through consensus between the 2 authors. In the second round of coding, each article was coded independently by both authors by applying the codebook. Then, the authors met weekly to discuss and resolve all discrepancies.

## Results

### Overview of the Included Articles

**Figure 1:**
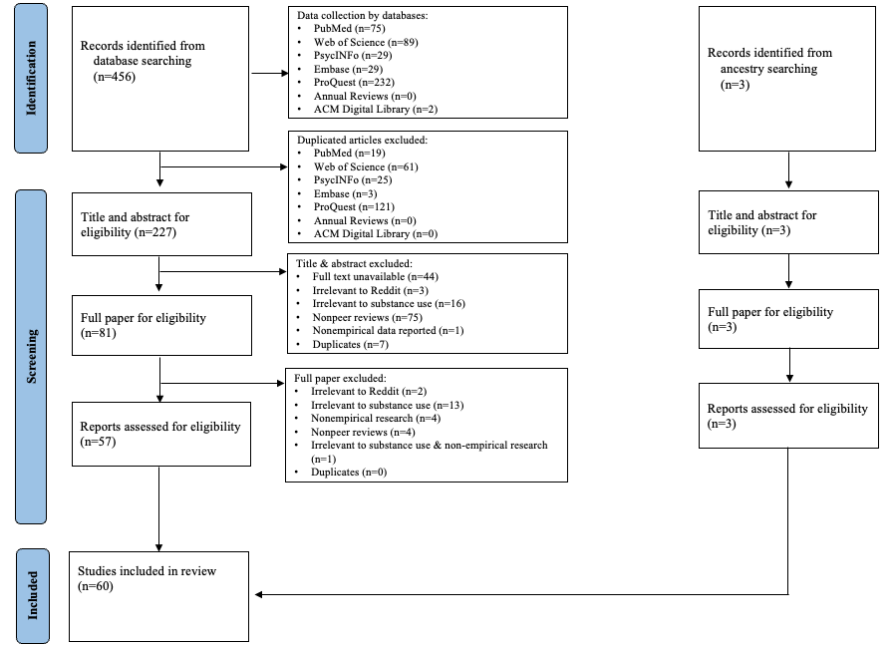
PRISMA (Preferred Reporting Items for Systematic Reviews and Meta-Analyses) flow diagram.

After screening, a total of 60 articles were included, with 57 articles identified from the initial database search and 3 from the ancestry search, which refers to the snowballing search conducted on the citations within the articles we initially included from databases and journals. Figure 1 presents the PRISMA (Preferred Reporting Items for Systematic Reviews and Meta-Analyses) flow diagram that outlines the screening and selection process for the included studies. Multimedia Appendix 2 contains the completed PRISMA checklist for reference. The included articles were published between 2015 and 2022, with the majority published in 2021 (22/60, 37%). In total, 82% (49/60) of the articles were published in journals, and the remaining 18% (11/60) of the articles were from conferences. *JMIR* (6/60, 10%) and *Drug and Alcohol Dependence* (6/60, 10%) were the top 2 journal venues for the included articles. The journals and conferences covered a broad range of areas, including humanities, medicine and health, biochemistry, computer science, mathematics, business and management, engineering, communication, pharmacology, toxicology and pharmaceutics, and psychology.

### Types of Substance Use

**Figure 2:**
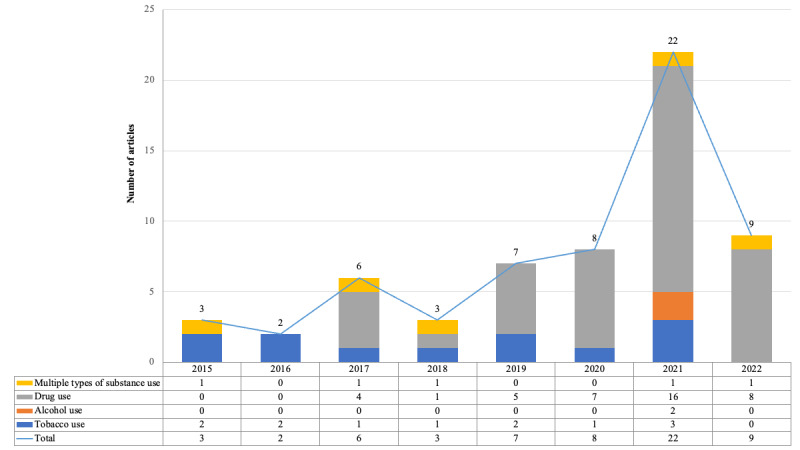
Trends of publications on different types of substance use for years.

We categorized substance use into 3 types: drug use, tobacco use, and alcohol use. Of the 60 included articles, 55 (92%) focused on 1 type of substance use, including drug use (n=41, 68%), tobacco use (n=12, 20%), and alcohol use (n=2, 3%). The remaining 8% (5/60) of the articles investigated multiple types of use, including tobacco and drug use (3/60, 5%), tobacco and alcohol use (1/60, 2%), and tobacco, alcohol, and drug use (1/60, 2%). The articles covered a broad range of substances, such as opioids (24/60, 40%), electronic cigarettes or vaping (10/60, 17%), marijuana (9/60, 15%), prescription psychotherapeutic drugs (3/60, 5%), hallucinogens (2/60, 3%), fentanyl (2/60, 3%), alcohol (2/60, 3%), cigarettes (1/60, 2%), cocaine (1/60, 2%), heroin (1/60, 2%), and kratom (1/60, 2%). Table S2 in Multimedia Appendix 1 provides details of the studies classified by type of substance use. In addition, Figure 2 displays the number of articles categorized by substance type and publication year. As illustrated in Figure 2, the use of Reddit data for studying substance use was first observed in 3 studies in 2015, with 2 focusing on tobacco use [18,45] and 1 examining both tobacco and alcohol use [19]. Subsequently, research on drug use on Reddit began in 2017 and has gained significant attention in the literature, reaching a peak in 2021. It is notable that the observed decrease in 2022 could be because of the timing of our literature search.

### Study Objectives (RQ1)

The objectives of the included articles are summarized into 5 themes (Table S3 in Multimedia Appendix 1). The most prevalent study objective was *to identify the trends and patterns of substance use discussions on Reddit* (52/60, 87%). Specifically, researchers aimed to examine trending topics, language features, and keywords related to substance use. Studies aligned with this objective have examined Reddit discussions on tobacco [63,64,66], alcohol [70], and drugs [28,49,59,75]. For example, Wang et al [18] classified popular e-cigarette flavors in Reddit discussions. In addition, Hu et al [60] summarized use patterns of cigarettes and vaping by analyzing discussions from 8 subreddit groups. Other studies have also aimed to identify Reddit users’ behavioral patterns, such as subjective experiences of microdosing [52,53] and self-help practices [36].

The second common study objective was *to explore individual characteristics of Reddit users who discuss substance use* (31/60, 52%). Studies with this aim have explored individual characteristics, including demographic information [32,47,50,52], emotions [46,50], motivations for substance use [24,68,73], motivations for treatment [29], and perceptions of substance use [45,62]. In addition, some studies have conducted surveys to better investigate Reddit users’ characteristics. For example, MacQuarrie and Brunelle [47] recruited users from Reddit, Facebook (Meta Platforms, Inc), and Twitter (Twitter, Inc) to examine the influence of individual characteristics, such as demographic variables and personality, on their perspectives regarding the decriminalization of drugs in Canada.

The third common study objective was *to propose or advance methodological approaches for analyzing Reddit data* (13/60, 22%). These approaches mainly involve automatic models that use machine learning and data mining techniques to analyze large-scale Reddit posts and comments related to substance use [10,36,55]. The fourth study objective was to *investigate interactions among Reddit users who discuss substance use* (6/60, 10%). These studies focused on various types of interactions, including information seeking in subreddits [57], information disclosure, social support [37,51,56], and social networking [23,33]. The last study objective was *to evaluate the effectiveness of health interventions and promotion campaigns delivered via Reddit* (1/60, 2%). One study identified this objective. Silberman and Record [65] explored the potential of Reddit as a platform for delivering health interventions on smoke-free policy compliance to college populations.

### Reasons and Limitations of Using Reddit (RQ2)

#### Reasons for Using Reddit to Study Substance Use

Out of the 60 studies included in this review, 52 (87%) cited at least 1 reason for using Reddit as a data source to study substance use. These reasons and studies are presented in Table 1.

**Table 1:** Reasons for using Reddit as a data source (N=60).

Reasons mentioned	Description	Studies, n (%)	References
Anonymous data	The anonymous nature of Reddit enables a more candid and open discussion of stigmatized topics.	26 (43)	[19-21,23,25,28-31,33,35,37-39,42,45,49,52-56,60,65,72,73]
Popularity of Reddit	Reddit is one of the most popular social networking sites.	24 (40)	[18,19,21-23,26-31,33,34,38,45,55,57,60,61,65,72-75]
Open and free platform	Reddit provides an open and free platform that enables researchers to examine discussions on stigmatized or sensitive topics among users.	22 (37)	[18,25,29-31,33-35,37,38,42,45,46,49,51,53-55,57,73-75]
Explicit topics (subreddits)	Discussions are organized into topics of interest (ie, subreddits), allowing researchers to identify people and topics of interest.	20 (33)	[19-23,25,28,30,44,46,48,55,57,59,60,63,66,70,71,73]
Original first-hand experience	Original and unfiltered user-generated posts provide first-hand user experiences.	20 (33)	[10,18,21,24,25,27,28,30-32,42,54,55,57,60,61,64,68,69,75]
Large-scale data sets	Large-scale data sets provide rich content and statistical power.	13 (22)	[27,34,37,38,48,49,52,54,55,62,66,74,75]
Ease of data collection	Reddit provides a public application programming interface for easier data collection.	9 (15)	[24-26,34,45,49,58,60,74]
Long-form posts	Each post contains up to 40,000 characters, providing rich contexts for analysis.	6 (10)	[21,24,25,45,58,61]
Upvotes and downvotes	Reddit users can engage on a post via upvoting and downvoting, allowing researchers to study trending topics.	5 (8)	[10,30,33,51,56]

The most frequently reported reason was the *anonymity of the Reddit data* (26/60, 43%). Usernames on Reddit are typically not associated with the user’s real identity unless the user voluntarily reveals it. This anonymity fosters an environment in which users feel comfortable discussing sensitive topics, such as substance use, and provides valuable insights for researchers [25,36]. Another commonly cited reason was the *popularity of Reddit* (24/60, 40%). Reddit’s popularity ensures that a wide range of users, including those who use substances, are represented in the data. In addition, Reddit is an *open and free platform* (22/60, 37%) where users can discuss substance use without fear of stigmatization or criminal repercussions, making it easier for researchers to access a diverse range of perspectives. Furthermore, the organization of discussions into *explicit topics or subreddits* (20/60, 33%) allows users and researchers to identify people and topics of interest [63,71]. The availability of *original first-hand experience* (20/60, 33%) from unfiltered user-generated posts has also emerged as a commonly cited reason. In addition, compared with other platforms, Reddit tends to have a lower amount of product advertisements and promotions and a higher proportion of user-generated discussions. As a result, it can be a valuable resource for gaining nuanced and contextualized insight into the opinions and experiences of users [24,64]. *Large-scale data sets* (13/60, 22%) provide rich content and statistical power, and Reddit also provides a public application programming interface (API) for *easier data collection* (9/60, 15%) of the *long-form posts* (6/60, 10%), offering rich contexts for analysis. Several studies have highlighted that a single post on Reddit can contain as many as 40,000 characters, which is significantly greater than Twitter’s character limit of 280. This characteristic of Reddit offers a wealth of in-depth content that can be analyzed [21,61]. Finally, the *upvote and downvote feature* (5/60, 8%) allows researchers to study trending topics and the opinions of the community [19,42].

#### Limitations of Using Reddit to Study Substance Use

Among the 60 articles reviewed, 19 (32%) did not report any limitations related to the data set collected from Reddit, whereas the remaining 41 (68%) articles mentioned at least 1 limitation owing to Reddit (Table 2).

**Table 2:** Limitations of using Reddit as a data source (N=60).

Limitations mentioned	Description	Studies, n (%)	References
Lack of representativeness of the general population with substance use	Reddit data may only represent a subsample of the population with substance use, skewing toward young, White, and male individuals in the United States who are prone to sharing and seeking information in online communities.	22 (37)	[10,17,19,20,23,25,28,29,31,33-35,40,42,44,52,53,60,62,72,74,75]
Lack of demographic information	There are limited data on users’ demographic information, such as age, sex, ethnicity, and geographic information.	21 (35)	[18,23-25,29,31,33,35,37,38,45,53,56,61,62,64,68,69,71-73]
Lack of longitudinal data	There are limited data on users’ substance use history or engagement beyond Reddit.	9 (15)	[19,23,28,35,42,59,60,63,64]
Unvalidated self-report data	Self-reported Reddit posts are not clinically verified and could contain second-hand experiences.	7 (12)	[28,30,38,45,49,59,73]
Reddit’s changing policies and nature limit the replicability	Reddit’s terms of service and the content of posts are constantly changing, thus affecting the replicability of the studies.	4 (7)	[33,59,65,72]
Challenges in analyzing long and unstructured posts	The lengthy (up to 40,000 characters) and free-flowing text posts are challenging to analyze.	3 (5)	[17,18,43]
Reddit’s restrictions on API^a^	Reddit imposes some constraints on the use of its official API; for example, up to 1000 posts can be retrieved at one time, and only the current badge of a user is accessible.	2 (3)	[19,46]

^a^API: application programming interface.

The *lack of representativeness of the general population with substance use* (22/60, 37%) was a commonly reported limitation. The data were potentially skewed toward young, White, and male individuals in the United States, who are more likely to seek and share information in web-based communities [19,36,60,62]. Furthermore*, the lack of demographic information available on Reddit users* (21/60, 35%) limited the analysis that could reveal substance use patterns based on demographic information, such as age, sex, ethnicity, and geographic location. For example, a study investigating the links between fentanyl, buprenorphine induction, and precipitated opioid withdrawal acknowledged that the absence of geographic information on Reddit users prevented them from determining whether individuals posting on Reddit were located in areas with a high fentanyl prevalence [31]. In addition, *the lack of longitudinal data* (9/60, 15%), such as users’ substance use history or engagement beyond their activity on Reddit, restricts the analysis of certain studies. The anonymity of Reddit posts poses difficulties in conducting longitudinal studies and monitoring the effectiveness of certain interventions [23,28]. Several studies also noted that *unvalidated self-report data* (7/60, 12%) are a limitation because posts on Reddit are not clinically verified and may contain second-hand experiences. Some studies reported that *Reddit’s changing policies and nature limit the replicability of studies* (4/60, 7%)*.* For example, Silberman and Record [65] were unable to replicate their study in which they posted smoke-free messages on college subreddits because of the policy change on Reddit. Although long-text posts are advantageous for examining complex perspectives and experiences, several studies have identified difficulties in *analyzing these lengthy and unstructured posts* (3/60, 5%). Finally, *Reddit imposes some restrictions on the use of its official API* (2/60, 3%), such as only allowing the retrieval of up to 1000 posts at 1 time and restricting access to certain user information.

### Methods of Using Reddit to Study Substance Use (RQ3)

#### Research Design

We first examined the overall research design of the studies: qualitative design, quantitative design, or mixed methods design. Most of the included articles used an exclusively quantitative design (37/60, 62%), and only 13% (8/60) of the articles adopted a qualitative design. Mixed methods design accounted for approximately one-quarter of the reviewed articles (15/60, 25%).

#### Data Collection Approaches

##### Data Collection From Reddit

All except 12% (7/60) of the included articles reported their approach to collecting Reddit data for their studies. We categorized these approaches into 1 of the 4 categories (Table S4 in Multimedia Appendix 1): *accessing publicly available Reddit data repository via APIs* (36/60, 60%)*, recruiting participants from Reddit* (7/60, 12%)*, manual Reddit data collection* (6/60, 10%)*, and web crawling Reddit data* (4/60, 7%).

In total, 60% (36/60) of the articles *collected Reddit data from publicly available repositories* and all but 5% (3/60) of the articles reported using 1 or multiple APIs to collect Reddit data. Of those that used APIs, 30% (18/60) of the studies used Reddit’s official API [76], often via the Python Reddit API Wrapper (Python Software Foundation) [77] and 2% (1/60) via R package (RedditExtractoR) [25]. Out of 60 studies, 13 (22%) used Pushshift [78], an archiving platform maintained by Jason Baumgartner [79], and 3 (5%) combined Pushshift with BigQuery (Google LLC) [80], a data warehouse managed by Google.

Out of 60 articles, 7 (12%) *used Reddit to distribute recruitment advertisements and recruit participants* for survey studies. Of these 7 studies, 3 (43%) exclusively used Reddit as their means of participant recruitment, whereas the remaining 4 (57%) studies posted recruitment advertisements on Reddit as well as other platforms such as Facebook, Twitter, MTurk (Amazon), or email lists of nonprofit organizations (eg, Smoke-Free Alternatives Trade Association and the American Vaping Association). Notably, studies that used multiple recruitment methods were able to identify the unique characteristics of Reddit users for substance use compared with other platforms. For instance, Saunders et al [44] reported recruiting the highest number of urban participants from Reddit compared with other recruiting platforms such as Facebook, AdWords (Google LLC), and MTurk.

Out of 60 articles, 6 (10%) *manually collected Reddit data using Reddit’s searching and ranking functions*. The data set size in these studies was relatively small. For example, Sharma et al [68] searched on Reddit’s own search engine using a combination of e-cigarette–related keywords and mental health–related keywords and ranked results by “relevance” and “all time.” As a result, they collected 3263 comments from 133 discussion threads for analysis. D’Agostino et al [33] manually collected the first 100 posts and their comments under the “hot” tab from a subreddit for opioid recovery and conducted a qualitative content analysis to reveal the themes.

The other 7% (4/60) of the articles reported *collecting Reddit data through web crawling or scraping*. One example is Chen et al [17], who used a web crawler, Wget, to aid in their Reddit data collection process.

##### Sources Other Than Reddit

Out of the 60 included articles, 48 (80%) adopted Reddit as the only source to collect data. Among the remaining 20% (12/60) of the articles that used multiple data sources, 13% (8/60) collected data from 3 other types of data sources: social media (eg, Facebook and Twitter) [20,26,55,58,61,70], open government data sets (eg, census data set and Centers for Disease Control and Prevention data set) [20,70], and web-based health communities (eg, Hookah Forum and Drugs-Forum) [17,26,27]. In addition, 7% (4/60) of the articles distributed surveys on Reddit and other online platforms, including Facebook [44,47,52,67], Twitter [47,52,67], e-cigarette discussion forums [67], AdWords [44], and MTurk [44].

##### Subreddits Used in Data Collection

**Figure 3:**
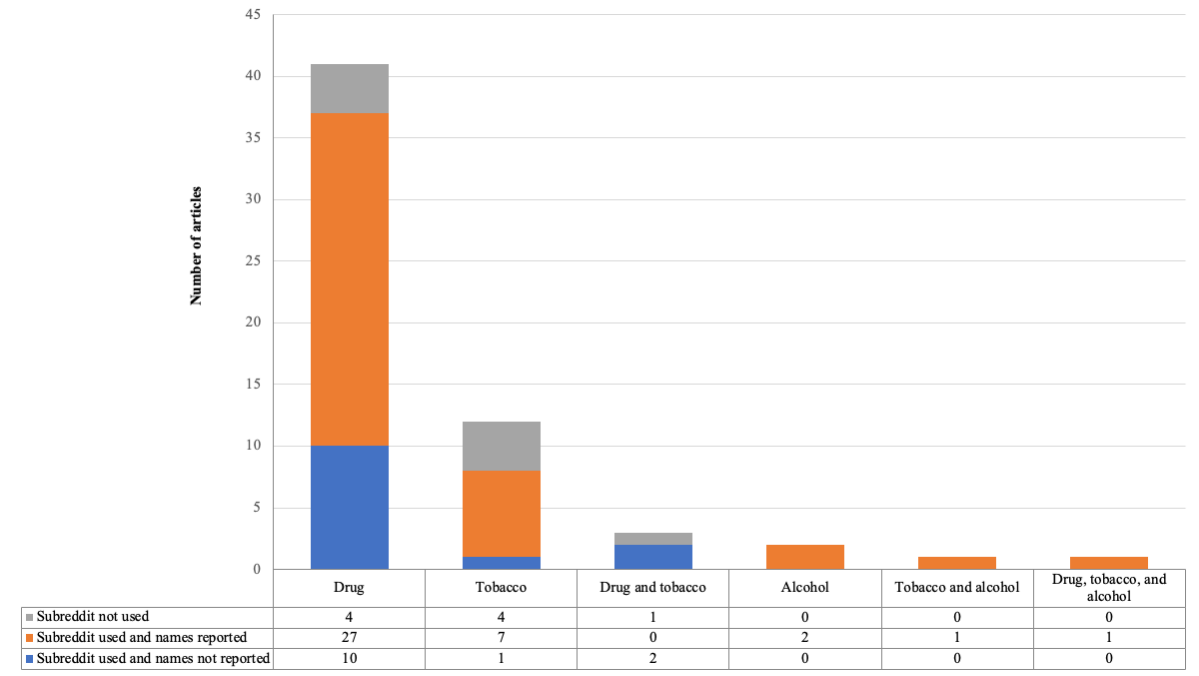
Subreddit use categorized by different types of substances.

We characterized whether the subreddit feature was used during the data collection process, and if used, whether the names of the subreddits were reported in the articles. Figure 3 presents the distribution grouped by articles studying different types of substances. Among all the 60 included articles, 51 (85%) took advantage of the forum-like subreddit feature for data collection, and 38 (63%) reported the names of the subreddits that had been used. The top 3 most adopted drug-related subreddits were r/opiates (14/60, 23%) [27,30-32,36,38,39,50,51,54-56,58,59], r/OpiatesRecovery (12/60, 20%) [10,30-32,36,38,39,46,50,51,54,58], and r/Drugs (6/60, 10%) [27,28,51,52,58,75]. The top 3 subreddits adopted for tobacco-related studies were r/stopsmoking (4/60, 7%) [19,45,60,63], r/electronic_cigarettes (4/60, 7%) [18,45,60,69], and r/vaping (3/60, 5%) [18,60,69]. Only 7% (4/60) of the included articles used subreddits to collect data for studying alcohol use, and they adopted subreddits such as r/StopDrinking, r/alcohol, and r/alcoholism. None of the subreddits were adopted by >1 included article for studying alcohol use. Of the 60 articles, 13 (22%) did not report from which specific subreddits they collected the data, although they mentioned using the subreddit feature to collect data. Notably, Chancellor et al [42] and Chen et al [17] attributed the reasons for hiding the subreddit names to ethical considerations. Instead of referring to the real names, they assigned fake names, such as OFFopiates [42] and QuitCannabis [17], to protect the anonymity of Reddit members.

#### Data Analysis Approaches

##### Unit of Analysis

Most of the included articles (44/60, 73%) chose individual posts, including initial posts (known as Reddit submissions) and replies to the posts (known as comments), as the unit of analysis. Only 13% (8/60) of the articles chose individuals or users as the unit of analysis. In total, 13% (8/60) of the articles analyzed both posts and individuals.

##### Annotation Approach

Most of the included articles (46/60, 77%) used annotation approaches to categorize posts or users, either manually or automatically. The annotation approaches were grouped into 3 main categories, including human annotation (34/60, 57%), Reddit’s existing labels (7/60, 12%), and automatic approach (11/60, 18%), as listed in Table 3. Some articles adopted multiple annotation approaches.

**Table 3:** Annotation approaches (N=60)^a^.

Category and subcategory			Studies, n (%)		References
**Human annotation (n=34)**					
	Researchers of the study	17 (50)		[20,24,25,28,31,33,34,45,53,57,59,60,65,68,69,73,75]	
	Clinically verified domain experts	7 (21)		[26,30,31,42,46,49,51]	
	Trained coders	6 (18)		[23,29,35,37,39,62]	
	Crowdsourced workers	1 (3)		[21]	
	Not explicitly reported	4 (12)		[18,36,48,66]	
**Reddit’s existing labels (n=7)**					
	Subreddits	5 (71)		[10,55,58,70,71]	
	Reddit’s badge	2 (29)		[19,63]	
**Automatic approach (n=11)**					
	Keyword matching or dictionary based	11 (100)		[17,28,36,37,39,50,61,64,66,71,72]	

^a^The numbers 34, 7, and 11 do not total 60 because not all of the 60 included articles used annotation approaches. Additionally, some articles might have used multiple annotation approaches.

1. *Human annotation*: (34/60, 57%): Most studies (17/60, 28%) relied on the researchers of the studies to perform the annotation without explicitly mentioning the researchers’ backgrounds. However, 12% (7/60) of the studies specified that the annotators were clinically verified domain experts. For instance, Andy and Guntuku [51] reported that their data were annotated by 3 health care professionals with graduate degrees and expertise in substance use. In total, 60% (6/10) of the studies trained the coders in coding sessions to ensure consistency and accuracy in the annotation process. For example, Brett et al [62] trained 6 coders for >12 hours of sessions, which included a review of the codebook, coding practice, disagreement resolution, and familiarization with the coding platform. Only 2% (1/60) of the studies employed crowdsourced workers, who were recruited from MTurk [21]. These workers were asked to code “yes” or “no” on Reddit posts regarding 2 questions: “May the user be at risk of suicide or intentional overdose?” and “Does the post imply opioid addiction?” Each post was annotated by 3 workers with a master’s degree who received monetary compensation and detailed coding instructions. Finally, 7% (4/60) of the articles mentioned human annotators but did not specify the coders’ backgrounds or domains of expertise.
2. *Reddit’s existing labels*: (7/60, 12%): Out of 60 articles, 7 (12%) made use of existing Reddit labels. In total, 8% (5/60) of these articles used subreddits as a means of weakly annotating topic categories. For instance, Lu et al [10] used posts collected from 2 drug use subreddits (ie, r/Opiates and r/Drugs) and 2 drug recovery subreddits (ie, r/OpiatesRecovery and r/RedditorsInRecovery) to train a classifier that could predict users’ transition from drug use to recovery. Similarly, Eshleman et al [58] used the fact that a user had posted in drug recovery–related forums as the ground-truth label in their study, which aimed to develop predictive models for identifying users who could benefit from recovery assistance. Another notable annotation approach was the use of Reddit “badges.” Certain subreddits, such as r/StopSmoking and r/StopDrinking, offer users the opportunity to earn “badges” that display the number of days they have remained abstinent. This “badge” information was used as a weak annotation of abstinence progress by 3% (2/60) of the articles included in the review [19,63].
3. *Automatic approach*: (11/60, 18%): Out of 60 articles, 11 (18%) used an automatic annotation approach, such as keyword- or dictionary-based matching, to annotate the Reddit posts. For example, in a study by Barker and Rohde [66], a dictionary comprising 7 e-cigarette topics was created and used to categorize relevant posts and to assess the prevalence of each topic on Reddit.

#### Algorithm Approaches

More than half of the included articles (35/60, 58%) used 1 or multiple algorithm approaches, which were classified into 4 categories (Table S5 in Multimedia Appendix 1): rule-based approach (21/35, 60%), traditional machine learning approach (18/35, 51%), neural network and deep learning approach (14/35, 40%), and graph network–based approach (3/35, 9%).

Rule-based algorithms were implemented in 60% (21/35) of the studies to recognize specific topics of interest, such as the mentioning of a certain substance or type of use in Reddit posts and comments. For example, Hu et al [60] developed an algorithm using regular expressions to distinguish between vaping cannabis and vaping nicotine and smoking cannabis and smoking nicotine. Machine learning approaches, including the traditional algorithms (eg, logistic regression, random forest, support vector machine, and naive Bayes) and neural network and deep learning algorithms (eg, convolutional neural networks and long short-term memory networks), are widely used for natural language processing tasks. Notably, topic modeling with latent Dirichlet allocation was adopted by 4 articles to uncover the underlying topics and themes from the free-flowing Reddit posts [38,45,56,74]. This approach allowed the researchers to identify popular topics and monitor how the topics changed with time in certain communities. For instance, El-Bassel et al [38] used latent Dirichlet allocation to identify the dominant themes in opioid-related subreddits during the initial 3 months of the COVID-19 outbreak, highlighting the need for attention from providers and policymakers on concerns related to access to medication for opioid use disorder. In total, 12% (7/60) of the articles used and compared multiple machine learning models to achieve the best performance. For example, Yao et al [21] trained a series of traditional and neural network text classifiers to identify suicidal ideation among opioid users and found that the convolutional neural network model performed the best. Similarly, Jha and Singh [50] used several machine learning models to identify misinformation about medication for opioid use disorder, with a logistic regression classifier combined with term frequency–inverse document frequency achieving the best performance.

Moreover, most studies that used machine learning approaches relied on supervised algorithms (16/25, 64%) rather than unsupervised approaches (9/25, 36%), except for Eshleman et al [58] who used both supervised and unsupervised algorithms (Table S6 in Multimedia Appendix 1). In total, 9% (3/35) of the articles used graph network–based approaches to explore the relationship between words [51], topics [66], or users [19]. Tamersoy et al [19] constructed a network of short-term and long-term abstainers to explore their interactions based on the postings and commenting behaviors in the subreddits.

### Implications and Contributions (RQ4)

#### Theoretical Implications

First, we examined the theoretical implications proposed among the 60 included articles. Only 8% (5/60) of the articles used theoretical frameworks to guide their research and proposed corresponding theoretical implications. The remaining 92% (55/60) of the articles did not incorporate any theory in their studies. Of the 5 studies that used theories, 3 (60%) studies used theories *to develop rationales for their hypotheses or research questions and discussed how their empirical findings supported the propositions of the theories* [37,65,66]. Specifically, Rhidenour et al [37] attributed the findings of the veterans’ support-seeking medical marijuana use on Reddit to the Social Identity Model of Deindividuation Effects theory [81], which proposes that a strong group identity, combined with anonymity, can increase liking and self-disclosure in web-based relationships, benefiting coping and health outcomes, especially for those facing social stigma. This finding not only supports the Social Identity Model of Deindividuation Effects theory but also adds to its understanding of web-based social support. Silberman and Record [65] developed their RQ about Redditors’ interaction with smoking-free campus policies through the lens of media system dependency theory [82]. Barker and Rohde [66] were guided by network theory [83] and social cognition theory [84] to investigate how Redditors discussed e-cigarettes within a network in a Reddit community.

The remaining 2 studies used theories *to develop codebooks for analysis* [23,42]. Bunting et al [23] adapted the Big Events framework [85] to guide their thematic analysis of the impact of COVID-19 on the social networks and social processes of people who use opioids, whereas Chancellor et al [42] adapted the transtheoretical model of behavior change [86] to develop a codebook for analyzing recovery among opioid users. These examples highlight the potential benefits of using theoretical frameworks to inform research on Reddit.

#### Methodological Implications

Out of 60 articles, 15 (25%) did not explicitly state their methodological implications. The remaining 75% (45/60) provided methodological implications, which can be classified into 3 categories: *proposing novel or advancing existing methodological approaches* (39/60, 65%), *validating existing methodological approaches* (11/60, 18%), *and providing open sources for future research (3/60, 5%*; Table S7 in Multimedia Appendix 1). Among the studies proposing novel or advancing existing methodological approaches, 40% (24/60) contributed to the development of classifications or codebooks, 20% (12/60) focused on developing models or algorithms, and 12% (7/60) focused on methodological designs and recruitment methods. In addition, 18% (11/60) of the studies focused on validating the existing methodological approaches on Reddit in the context of substance use. Notably, only 5% (3/60) of the studies provided future researchers with open sources such as original computational codes [60], annotated data sets [46], and a publicly available application [32].

#### Practical Implications

We categorized the reported practical implications from the articles into 5 groups: *Reddit discussions on substance use* (57/60, 95%)*, recommendations for clinical practices and policies* (43/60, 72%)*, comparison of Reddit discussions on substance use across various sources* (30/60, 50%)*, privacy concerns on using Reddit data for substance use research* (4/60, 7%)*, and development of novel applications* (2/60, 3%; refer to Table S8 in Multimedia Appendix 1 for a detailed description and corresponding references).

#### Reddit Discussions on Substance Use: Topics, Factors, and User Characteristics

Practical implications related to Reddit discussions about substance use (57/60, 95%) were further categorized into three main themes:

1. *Trending topics about substance use*: (43/60, 72%): Trending topics were revealed in these studies, including popular subreddits [31], linguistic features of the discussions [63], popular e-cigarette or e-liquid flavors [18,61,64,69], commonly mentioned types of substances [41,49], and exchange of social support related to substance use [23,51].
2. *Factors associated with substance use*: (26/60, 43%): These studies examined the associations between substance use and various factors. For example, Smith et al [25] identified a significant correlation between kratom use and kratom addiction, showing that higher doses of kratom are more likely to lead to addiction.
3. *User characteristics*: (21/60, 35%): A total of 21 studies focused on user-centered analysis to classify the characteristics of Reddit users who engage in discussions about substance use, such as identifying the motivations for substance use [37,53,73], barriers to microdosing and vaping practice [53], and adverse effects and withdrawal symptoms experienced by individual users [25].

#### Recommendations for Clinical Practices and Policies

Practical implications including recommendations (43/60, 72%) were categorized into two types:

1. *Recommendations for clinical practices*: (35/60, 58%): A total of 35 studies provided ways to advance future health campaigns, interventions, and treatments through clinical practices. Overall, studies suggest that Reddit is a promising platform in the context of substance use and recovery from several perspectives. For substance-related health campaigns, exploring Reddit discussions can help campaigners better understand their target audiences, address misinformation related to their target health issues, and identify social influencers to promote healthy behaviors [26]. In addition, the anonymous features of Reddit encourage users to disclose and seek support without releasing their identifiable information, allowing clinicians and physicians to facilitate conversations about interventions and treatment for recovery [19,21,28].
2. *Recommendations for substance-related policies*: (20/60, 33%): A total of 20 studies provided recommendations for policy makers to facilitate the effectiveness of substance-related regulation. Reddit provides policy makers with a tool to collect trending public opinions on a large scale, including concerns and potential factors that can affect the effectiveness of substance-related regulation [29,34]. By gathering ideas from public opinions, decision makers can improve their understanding of the needs and perspectives of different stakeholders and ultimately develop more effective policies.

#### Comparison of Reddit Discussions on Substance Use Across Various Sources

In total, 50% (30/60) of the studies provided practical implications by comparing Reddit discussions across four types of sources:

1. *Comparison over time*: (17/60, 28%): A total of 17 studies included a temporal analysis to reveal the changing patterns in Reddit discussions over time. For example, Sarker et al [34] collected Reddit data from both pre–COVID-19 and COVID-19 periods. Their results indicated a peak in discussions about treatment at the beginning of the pandemic.
2. *Comparison across various substances*: (17/60, 28%): A total of 17 studies revealed the frequencies of use of various types of substances based on Reddit discussion [34,60,74]. For example, Hu et al [60] compared the frequencies of use of tobacco, cannabis, and vaping and cessation mentioned by Reddit users.
3. *Comparison across diverse user groups*: (11/60, 18%): A total of 11 studies compared different user groups based on demographics [40], geodemographics [44,70], substance dose use [22,52], substance use history [19,32,67], and withdrawal experience [63,73].
4. *Comparison across different social media platforms*: (4/60, 7%): A total of 4 studies compared Reddit with other social media platforms, including Twitter [26,70], YouTube (Google LLC) [26], Vapor Talk [17], and Facebook [44]. These studies revealed different trending topics from different social media platforms.

#### Privacy Concerns About Using Reddit Data for Substance Use Research

Out of 60 studies, 4 (7%) highlighted privacy concerns related to using Reddit data for substance use research. The anonymity of Reddit allows researchers to access their data without the approval of an ethics board. However, although this feature safeguards Reddit users from being recognized by researchers and other users, those who have shared their substance use history and experiences on Reddit may be identified by others. Such identification can result in negative consequences for these individuals, including potential damage to their reputation, career, and even investigation of crimes [42]. In addition, Reddit is a public domain that lacks password protection; therefore, users should be mindful of its public nature while engaging with others on the web [49]. Thus, researchers must carefully consider the ethical implications of using Reddit data for substance-related research [42,49,55,75].

#### Development of Novel Applications

In total, 3% (2/60) of the studies developed and tested new platforms, software, or devices for substance users. Preiss et al [36] developed a web application for researchers to identify substance-related symptoms and treatments. Moghadasi et al [43] developed a chatbot that can answer questions about substance use and addiction. These studies used Reddit data to train and enhance their models.

## Discussion

### Principal Findings

#### Trends and Topics of Using Reddit to Study Substance Use

The use of Reddit as a data source for studying substance use has increased steadily since 2015, with a sharp increase observed in 2021. In terms of the study objective, the most common objective in studies using Reddit to study substance use was to identify trends and patterns in various types of substance use discussions (52/60, 87%). Apart from it, researchers have been using Reddit to explore the unique experiences and perspectives of users; propose and advance methodological approaches, especially automatic models; investigate interactions among users; and develop interventions to address substance abuse. This trend can be attributed to various factors, including the growing use of Reddit, changes in the prevalence and types of substance use over time, the increasing accessibility of Reddit data, the development of various tools and techniques for analyzing Reddit data, and the impact of the COVID-19 pandemic.

The growing use of Reddit has provided researchers with a wealth of data on substance use. The number of monthly active Reddit users worldwide has increased by 618% from approximately 120 million in 2015 to 861 million in 2021 [87], contributing to the rise in studies using Reddit to study substance use. In addition, changes in the prevalence and types of substance use over time may also explain the trend in the literature. For example, the opioid crisis was declared a national public health emergency in the United States on October 26, 2017 [56], and the ongoing opioid epidemic has led to a significant increase in research on opioid use and related issues. Correspondingly, the literature on Reddit reflects this trend, with 24 opioid-related studies identified in this review. Similarly, the growing legalization of cannabis in many countries may have led to an increase in the research on cannabis use. In addition, the accessibility of Reddit data has played a role in the rise of studies using Reddit to study substance use. Since 2018, leading social media platforms such as Facebook, Instagram, and Twitter have started to limit their API access because of scandals around data privacy and ethics [88,89]. In contrast, Reddit’s API remains open and free. The availability of Reddit’s open API has enabled researchers to collect large amounts of data without violating user privacy, with more than half of the studies (36/60, 60%) in this review using APIs to collect Reddit data. The large-scale Reddit data set has also led to the development of various tools and techniques for analyzing social media data. We identified 22% (13/60) of the studies with the study objective of proposing or advancing methodological and analytical approaches for analyzing Reddit data. These studies demonstrated the potential of using machine learning and data mining techniques to analyze large-scale Reddit posts and comments related to substance use. Finally, the impact of the COVID-19 pandemic may have led to a sharp increase in studies using Reddit to study substance use in 2021. The pandemic has brought significant changes to daily life, including changes in substance use patterns and behaviors. In total, 12% (7/60) of the articles that used Reddit to study the impact of the pandemic on people with substance use were identified in this review [20,30,34,36,39,46,75].

#### Strengths and Limitations of Using Reddit Data for Substance Use Research

Our second RQ explored the reasons and limitations of using Reddit data from the 60 included research articles. The unique features of Reddit, including anonymity, original first-hand experience, large-scale data sets, long-form posts, explicit topics (ie, subreddits), ease of data collection, and the upvoting and downvoting reactions to the posts, make it a valuable resource for researchers who are interested in studying sensitive topics that users may not be willing to discuss openly, such as substance use, mental health [6,90], and sexual abuse [91]. However, the review also identified several limitations of using Reddit data, especially in the context of substance use research. First, the low representativeness of the general population is a significant concern mentioned in 37% (22/60) of the articles, and this limits the generalizability of the findings to the substance use population beyond Reddit users. Reddit users, often skewed toward younger and more technologically adept male individuals [92], might not mirror the diversity and lived experiences of the larger substance use population. Researchers aiming to study diverse demographic groups should be mindful of this limitation. Second, the lack of demographic information and limited data on users’ substance use history make it challenging to identify and control certain variables, such as location, sex, and use history, and further deploy interventions. Such gaps can impede the comprehensive analysis of user patterns and hinder the customization of interventions targeting specific user groups. In addition, self-reported data without clinical verification may introduce bias and raise questions on the studies’ reliability. This limitation may lead to inaccuracies in the reported symptoms and social desirability bias, where users might portray themselves more favorably [93]. Finally, the unstructured nature of the posts presents challenges to analyzing them, especially when dealing with large-scale data sets. Advanced computational linguistic tools and methodologies are usually required to parse through unstructured texts to obtain meaningful insights [94]. Researchers who solely rely on Reddit data to study substance use should acknowledge these limitations and cautiously interpret the findings and propose implications.

#### Methodological Approaches of Using Reddit Data for Substance Use Research

In terms of the research design adopted in the articles exploring substance use on Reddit, the majority used an exclusively quantitative approach (37/60, 62%). This finding aligns with the results of a recent study by Proferes et al [12], which found that 66.4% of 727 reviewed manuscripts using Reddit as a data source used a quantitative research design. However, the number of studies using qualitative approaches in this review (8/60, 13%) was much lower than that identified by Proferes et al [12] (25.2%). This disparity suggests a lack of qualitative research on substance use on Reddit, highlighting the need for further exploration of this research approach in this context.

Data collection approaches were classified into 4 categories: accessing publicly available Reddit data repositories via APIs, recruiting participants from Reddit, manual Reddit data collection, and web crawling Reddit data. The use of APIs was the most common approach for data collection in the included articles, and >50% of the studies used APIs to collect Reddit data. Notably, apart from the official API, Pushshift, a platform developed by Jason Baumgartner in 2015 [79], was widely used in 22% (13/60) of the articles in this review. Although historically valued for its access to extensive Reddit data and larger querying limits compared with Reddit’s official API [6,12], recent changes have dramatically altered the landscape of Pushshift’s availability and use. On April 18, 2023, Reddit announced a series of changes to its API terms [95]. These changes not only resulted in charging commercial entities requiring large-scale data access but also identified Pushshift as noncompliant with its new terms. Consequently, as of May 2023, Pushshift’s data API access was revoked [96]. Although partially restored for subreddit moderators, its utility for general research purposes remains limited as of June 30, 2023 [97,98]. Such restrictions highlight the increasing challenges researchers face in accessing large-scale data from platforms such as Reddit, echoing the similar constraints seen on other platforms such as Facebook and Twitter [12,99]. This new paradigm underscores the fragile reliance on third-party tools and the necessity of forging more consistent data access pathways to ensure the robustness of psychosocial research on substance use and recovery.

Most studies (44/60, 73%) chose individual posts or comments as the unit of analysis, and most studies (46/60, 77%) used annotation approaches to categorize posts or users either manually or automatically, with human annotation being the most common approach (34/60, 57%). Overall, >50% of the included articles used 1 or multiple algorithm approaches. Consistent with the results of methodological reviews of social media data analysis [6,100], we found that machine learning approaches, including the traditional algorithms and neural network and deep learning algorithms, are widely used for natural language processing tasks.

#### Theoretical, Methodological, and Practical Implications of Using Reddit Data for Substance Use Research

Our final RQ aimed to explore the implications of using Reddit data to investigate substance use from theoretical, methodological, and practical perspectives. The findings indicate that theoretical frameworks were used in only 8% (5/60) of the studies to guide their research and propose corresponding theoretical implications. Notably, each study used different theories, highlighting the need for further research on the benefits of incorporating theoretical frameworks in this area.

In total, 77% (46/60) of our reviewed articles explicitly included methodological implications, with most studies (39/60, 65%) proposing novel or improved existing methodological approaches. These findings can contribute to the development of more accurate and efficient methods for analyzing Reddit data on substance use. Of the 60 studies, only 3 (5%) provided open-source contributions, with 1 (2%) providing the original source code [60], 1 (2%) sharing annotated data set [46], and 1 (2%) publishing a public application [32]. Given the potential benefits of open source and data sharing, the finding highlights the need for promoting open-source practice efforts, such as incentives, policies, and data sharing training and advocacy programs [101,102]. It is also important to note that 25% (15/60) of the studies did not explicitly state their methodological implications, which suggests a potential gap in the reporting of research methods in this area. To ensure the rigor and reproducibility of future research, it is essential for researchers to clearly state their methodological implications.

In addition, all studies explicitly discussed the practical implications. The most prevalent practical implications related to Reddit discussions about substance use are the investigation of trending topics, such as linguistic features of the discussions, and the commonly mentioned types of substances. Examining such conversations and topics can provide valuable insights into emerging trends and potential public health concerns. For example, Barenholtz et al [27] revealed that an increase in Reddit mentions of 7 novel psychoactive substances was soon followed by a corresponding increase in toxicology positivity, highlighting the significance of Reddit data in informing public health interventions and policies aimed at addressing substance use. Therefore, the information gleaned from analyzing Reddit data can be useful for researchers, clinicians, and policy makers seeking to understand the current state of substance use discussions on the platform.

### Gaps and Future Directions

#### Strengths and Limitations of Anonymity

Anonymity has double-edged effects on the research of stigmatized topics. Anonymity is a unique feature of Reddit that distinguishes it from other social media platforms. This distinctive feature has been widely discussed in our included research papers [23,38,53]. Anonymity can promote open discussion on Reddit and enable Reddit users to share experiences on sensitive topics more freely, thus allowing researchers to gain valuable insights. However, anonymity also poses several limitations. First, anonymity hinders researchers from identifying user-level information, such as demographics, geographics, users’ substance use history, or Reddit use data [36,42,73]. Lacking these pieces of information could limit the scope of the investigations and make the context of the findings unclear. For example, geographics can be a critical factor because regulations and trending substances vary by location [31,38,72,73]. Time can be another critical factor because concerns about opioid use have been exacerbated during the COVID-19 pandemic [34]. In addition, anonymity challenges researchers who aim to track specific Reddit users for longitudinal behavioral data. Moreover, it is challenging to verify the data accuracy from Reddit as these self-reported messages are clinically validated [38,65,73]. Finally, Reddit data from specific subreddits may not be representative of the entire population of substance users because Reddit users are predominantly identified as young male adults [98]. To address these limitations, future studies are encouraged to consider these strengths and limitations of anonymity while using Reddit data to explore web-based discussions about substance use.

#### Ethical Considerations for Future Studies of Substance Use on Reddit

Among our included articles, only a few discussed ethical issues while using Reddit data to explore substance use. Although Reddit data are open and public and the anonymity offered by Reddit discussions enables a more honest sharing of views, which is crucial for research on sensitive topics, the potential harm to research participants should not be overlooked, especially considering the sensitive nature of the research topics and the susceptibility of the population being studied.

Moving forward, researchers must be aware of the ethical considerations involved and take appropriate measures to ensure the protection of research participants. Future studies could focus on developing guidelines for the use of Reddit data in sensitive topics such as substance use research. As discussed by Proferes et al [12], there remains a lack of uniformity in the current ethical practices among researchers using public data from Reddit. The results of their systematic analysis of 727 Reddit studies indicated that only 101 (13.9%) studies explicitly mentioned “IRB” or ethical review. In addition, the use of Reddit data has raised several ethical issues that researchers should consider, such as defining the concept of “public,” determining the need for safeguarding data source identities, and establishing the level of anonymity required for research participants. Therefore, the guidelines for ethical practices using Reddit data should consider the privacy and confidentiality of research participants, the potential for harm, and the appropriate use of pseudonyms. We observed that several studies used pseudonyms instead of real subreddit names, which could be a good practice [17,42]. Moreover, we suggest that better deidentification of sensitive and personal data be used to ensure that research participants remain anonymous, and their privacy is protected.

#### Opportunities for Theoretical Development and Exploration

Our analysis of empirical research on Reddit indicates a lack of theoretical incorporation into most studies examining substance use. This finding underscores the need for further theoretical development and exploration in this area. Our findings are consistent with previous systematic review studies that also revealed a lack of theory-driven studies using computational and quantitative methods [100,103].

We encourage researchers to consider theoretical frameworks in their studies on substance use on Reddit. The incorporation of theoretical implications would not only enhance the quality of the research but also provide a better understanding of the underlying mechanisms of substance use on Reddit. Further theoretical development and exploration will also lead to the identification of new RQs and hypotheses that can be tested in future studies. Ultimately, the integration of theoretical perspectives in empirical research on Reddit will improve our understanding of substance use and provide a basis for developing effective interventions to address this growing public health concern.

#### Beyond Descriptive Patterns: Moving Toward Causality and Intervention Studies on Reddit

The most common study objective was to identify trends and patterns regarding the Reddit discussions of substance use. Although these studies provide insight into the topics discussed on Reddit and the characteristics of Reddit users in the context of substance use, they also raise questions about the root causes of trending patterns and the factors that drive individuals toward recovery, such as motivations to begin the recovery stages [37,53,73], barriers during the recovery practices [53], and the adverse effects of withdrawal experience [25]. Although quantitative designs were predominant in our included articles, we encourage future studies to include diverse methods and explore why the trends occur in specific subreddits. For example, conducting in-depth interviews with Reddit users who participated in substance use discussions could provide valuable user-level information [40]. In addition, there is a clear need to apply these findings in clinical or behavioral contexts. Approaches might involve clinical trials comparing Reddit use with nonuse in addiction recovery or understanding how platform algorithm adjustments can encourage healthier behavior and outcomes [104,105]. Potential interventions might explore just-in-time peer or moderator interventions or offer structured approaches to Reddit engagement for addiction recovery.

In addition, future studies using Reddit data may build on the previous results to explore the contextual factors leading to such trends or patterns in the discussions of substance use. Specifically, we encourage future researchers to compare linguistic features across various languages or different countries to enquire whether geographic information is a crucial factor in different patterns of linguistic features. Researchers can also compare different time points to examine whether these linguistic features differ across specific time points. Future studies examining associations across factors will contribute to deeper insights into the practical implications of substance use.

### Limitations

This review has several limitations. First, despite our comprehensive literature search across 7 databases using various keyword combinations, there remains a possibility that some literature on newly developed substances may have been overlooked. Second, we did not include a step of study quality assessment [106] during the screening process. This was because all the included articles were published in peer-reviewed journals or conferences, indicating good quality. In addition, given our objective of providing a comprehensive overview of substance use research on Reddit, we aimed to cover a broad range of literature. However, we acknowledge that the absence of a quality assessment may have given equal weight to all included studies, regardless of their quality, which should be considered. Future reviews may consider incorporating study quality assessment as part of their screening process.

### Conclusions

This systematic scoping review is the first to provide a landscape overview of using Reddit as a data source for studying substance use. Reddit has become a popular platform for exploring trends, patterns, and user experiences related to substance use discussions. Although the limitations of Reddit data must be considered, the information gleaned from analyzing Reddit data can be useful for researchers, clinicians, and policy makers seeking to understand the current state of substance use discussions on the platform and to develop effective interventions and policies. Our review also highlights gaps in the literature and suggests avenues for future research, such as the strengths and limitations of anonymity, ethical considerations, theoretical development, and moving beyond descriptive patterns. Overall, this review contributes to a better understanding of the potential and challenges of using Reddit as a data source for substance use research.

## Appendix

Multimedia Appendix 1 Supplementary tables.

Multimedia Appendix 2 PRISMA (Preferred Reporting Items for Systematic Reviews and Meta-Analyses) checklist.

## Abbreviations

API: application programming interface
PRISMA: Preferred Reporting Items for Systematic Reviews and Meta-Analyses
PRISMA-ScR: Preferred Reporting Items for Systematic Reviews and Meta-Analyses extension for Scoping Reviews
RQ: research question
SUD: substance use disorder

## Data Availability

All data generated or analyzed during this study are included in this published paper and its supplementary information files.
