# Supplementary material for: Investigating Substance Use via Reddit: Systematic Scoping Review
Source: J Med Internet Res. 2023 Oct 25;25:e48905. doi: 10.2196/48905 (PMC10637357; doi:10.2196/48905)
Supplement: Multimedia Appendix 1 [file jmir_v25i1e48905_app1.docx]

**Multimedia Appendix 1**

*Appendix Table* 1. Database Search Strategies

| Database | Search fields | Search strategy |
| --- | --- | --- |
| **PubMed** | Title/Abstract | (substance OR drug OR opioid OR opiate OR marijuana OR cannabis OR alcohol OR drinking OR tobacco OR smoking) AND (reddit) |
| **Web of Science** | Title,  or  Abstract | AB=((substance OR drug OR opioid OR opiate OR marijuana OR cannabis OR alcohol OR drinking OR tobacco OR smoking) AND (reddit))    TI=((substance OR drug OR opioid OR opiate OR marijuana OR cannabis OR alcohol OR drinking OR tobacco OR smoking) AND (reddit))    (substance OR drug OR opioid OR opiate OR marijuana OR cannabis OR alcohol OR drinking OR tobacco OR smoking) AND (reddit) (Title) or (substance OR drug OR opioid OR opiate OR marijuana OR cannabis OR alcohol OR drinking OR tobacco OR smoking) AND (reddit) (Abstract) |
| **PsycINFO** | Title/Abstract | TI ( (substance OR drug OR opioid OR opiate OR marijuana OR cannabis OR alcohol OR drinking OR tobacco OR smoking) AND (reddit) ) OR AB ( (substance OR drug OR opioid OR opiate OR marijuana OR cannabis OR alcohol OR drinking OR tobacco OR smoking) AND (reddit) ) |
| **EMBASE** | Title/Abstract | (substance:ab,ti OR drug:ab,ti OR opioid:ab,ti OR opiate:ab,ti OR marijuana:ab,ti OR cannabis:ab,ti OR alcohol:ab,ti OR drinking:ab,ti OR tobacco:ab,ti OR smoking:ab,ti) AND reddit:ab,ti |
| **ProQuest** | Title/Abstract | ab((substance OR drug OR opioid OR opiate OR marijuana OR cannabis OR alcohol OR drinking OR tobacco OR smoking) AND (reddit)) OR ti((substance OR drug OR opioid OR opiate OR marijuana OR cannabis OR alcohol OR drinking OR tobacco OR smoking) AND (reddit)) |
| **Annual Reviews** | Title/Abstract | (substance OR drug OR opioid OR opiate OR marijuana OR cannabis OR alcohol OR drinking OR tobacco OR smoking) AND (reddit) |
| **ACM Digital Library** | Title/Abstract | [[Title: substance] OR [Title: drug] OR [Title: opioid] OR [Title: opiate] OR [Title: marijuana] OR [Title: cannabis] OR [Title: alcohol] OR [Title: drinking] OR [Title: tobacco] OR [Title: smoking]] AND [Title: reddit] AND [[Abstract: substance] OR [Abstract: drug] OR [Abstract: opioid] OR [Abstract: opiate] OR [Abstract: marijuana] OR [Abstract: cannabis] OR [Abstract: alcohol] OR [Abstract: drinking] OR [Abstract: tobacco] OR [Abstract: smoking]] AND [Abstract: reddit] |

*Appendix Table 2 Types of Substance Studied*

| Type of Studies | Types of substance use | Number of articles (N=60, %) | Sources |
| --- | --- | --- | --- |
| **Studies focused on one type of substance use** | Drug use | 41 (68.3%) | [10,20-59] |
|  | Tobacco use | 12 (20%) | [17, 18,60-69] |
|  | Alcohol use | 2 (3.3%) | [70,71] |
| **Studies focused on multiple types of substance use** | Tobacco and drug use | 3 (5%) | [72-74] |
|  | Tobacco and alcohol use | 1 (1.7%) | [19] |
|  | Tobacco and alcohol and drug use | 1 (1.7%) | [75] |

*Appendix Table 3* Study Objectives

| Study Objective | Description | Number of Studies (N=60) | Sources |
| --- | --- | --- | --- |
| **To identify the trends and patterns of substance use discussions on Reddit** | Studies aim to identify the prevalence, timing, and content of discussions on various types of substances, such as popular flavors of e-cigarettes, and micro-dosing practice. | 52 | [10,17-20,22-33,35-42,44-46,48,49,52-56,58-64,66-75] |
| **To explore individual characteristics of Reddit users who discuss substance use** | Studies aim to explore individual characteristics, such as self-reported demographic information, emotions, and motivations. | 31 | [19,22-24,29,31-35,38-40,42,44-48,50,52,53,56,58,61,62,68,69,72-74] |
| **To propose or advance methodological/analytical approaches for analyzing Reddit data** | Studies aim to propose a new approach or advance an existing one, such as algorithms, statistical models, or other techniques. | 13 | [10,21,26,27,36,39,41,43,54,55,58,70,71] |
| **To investigate the interaction among Reddit users who discuss substance use** | Studies aim to investigate the nature of interactions between Reddit users, such as social networking, and seeking and providing support. | 6 | [23,33,37,51,56,57] |
| **To evaluate the effectiveness of health interventions and promotion campaigns delivered via Reddit** | Studies aim to evaluate the effectiveness of interventions and health promotion campaigns in reducing substance use or promoting healthy behaviors when implemented on Reddit | 1 | [65] |

*Appendix Table 4* Data Collection from Reddit

| Data Collection Approach | | Number of Studies (N=60) | Sources |
| --- | --- | --- | --- |
| **Accessing publicly available Reddit data repository via APIs** | | 36 |  |
|  | Reddit’s API | 17 | [19,23,25,27-31,34,35,37,43,45,46,50,56,58] |
|  | Pushshift | 9 | [10,49,55,60-62,64,66,70] |
|  | Pushshift and Google’s Big Query | 3 | [36,41,73] |
|  | Google’s Big Query | 2 | [42,51] |
|  | Reddit’s API and Pushshift | 1 | [21] |
|  | An API developed by Reddit users and hosted in Github^a^ | 1 | [75] |
|  | API not specified | 3 | [54,72,74] |
| **Recruiting participants from Reddit** | | 7 |  |
|  | Distributed recruitment advertisements on Reddit exclusively | 3 | [22,32,40] |
|  | Distributed recruitment advertisements on Reddit along with other platforms | 4 | [44,47,52,67] |
| **Manual Reddit data collection** | | 6 |  |
|  | Via Reddit’s search and ranking functions | 6 | [20,33,53,59,65,68] |
| **Web crawling Reddit data** | | 4 |  |
|  | Simply mentioning crawling Reddit data | 4 | [17,39,57,63] |
| **Not reported** | | 7 |  |
|  | Details of data collection approach unclear | 7 | [18,24,26,38,48,69,71] |

^a^ This API is no longer available at the time of the current study (https://github.com/camas/reddit-search/)

*Appendix Table 5 Algorithm approaches*

| Algorithm Approach | Number of Included Studies (N=35) | Sources |
| --- | --- | --- |
| **Rule-based** | 21 | [17,19,27,28,31,36,37,39,50,51,54-56,58,60,61,64,66,72-74] |
| **Traditional Machine Learning** | 18 | [10,17,19,21,26,30,34,38,39,42,46,51,56,58,60,63,66,74] |
| **Neural Network and Deep Learning** | 14 | [21,26,30,36,39,41-43,49,55,58,60,70,71] |
| **Graph-Network-based** | 3 | [19,46,66] |

*Appendix Table 6 Machine Learning Model Type*

| Model Type | Number of Included Studies (N=25) | Sources |
| --- | --- | --- |
| **Supervised** | 16 | [19,21,26,30,36,39,42,46,49,51,55,58,60,63,70,71] |
| **Unsupervised** | 8 | [17,34,38,41,43,56,66,74] |
| **Both Supervised and Unsupervised** | 1 | [10] |

*Appendix Table 7 Methodological Implications*

| Categories | Number of Included Studies (N=60) | Sources |
| --- | --- | --- |
| **Proposing novel or advanced existing methodological approaches** | 39 |  |
| Classifications/codebook | 24 | [18,23-26,30,31,35,36,39,42,45,46,48,51,53,57,59,61,65,66,67,69,73] |
| Models/algorithms | 12 | [19,26,41,43,46,49,50,54,55,58,70,71] |
| Methodological design/recruitment methods | 7 | [22,28,29,33,44,45,58] |
| **Validating existing methodological approaches on Reddit** | 11 |  |
| Models/algorithms | 8 | [21,26,50,51,60,63,65,68] |
| Methodological design | 2 | [32,75] |
| **Providing open sources for future research** | 3 |  |
| Original computational codes | 1 | [60] |
| Annotated datasets | 1 | [46] |
| Publicly available applications | 1 | [32] |
| **Not reported explicitly methodological implications** | 15 | [10,17,20,27,34,37,38,40,47,52,56,62,64,72,74] |

*Appendix Table 8 Practical Implications*

| Categories | Number of Included Studies (N=60) | Sources |
| --- | --- | --- |
| **Reddit discussions on substance use** | 57 |  |
| Topics related to substance uses | 43 | [17-21,23,24,25,27,30-36,38-42,48,49,51,53-61,63-66,68,69,72-75] |
| Factors related to substance use | 26 | [22,23,25-27,29,31-33,40,44,45,47,51,52,56,58,63,64,66-71,73] |
| Characteristics of users | 21 | [10,17,22,23,25,28,33,35,37,45,48,53,62,64,65,67,69,71-73,75] |
| **Recommendations for clinical practices and policies** | 43 |  |
| Recommendations for clinical practice, such as interventions and campaigns for promoting health outcomes, or strategies for supporting recovery. | 35 | [10,17,19,21,23,24,26,28-30,32-39,42,44-46,49-54,57-59,65,68,72,74] |
| Recommendations for substance-related policies | 20 | [18,19,28,30,33-35,37-39,41,45,47,48,54,61,63,67,69,72] |
| **Comparisons of Reddit discussions on substance use across various sources** | 30 |  |
| Comparisons over time | 17 | [18,24,27,31,33,34,38,40,41,54,56,61,64,67,72-74] |
| Comparisons across various substances | 17 | [22,24,25,27,31,33,34,40,41,45,52,56,58-60,72,74] |
| Comparisons across diverse user groups | 11 | [19,22,29,40,44,52,56,63,67,70,73] |
| Comparisons across social media platforms | 4 | [17,26,44,70] |
| **Privacy concerns on using Reddit data for substance use research** | 4 |  |
| Privacy concerns while using Reddit data in research | 4 | [42,49,55,75] |
| **Development of novel applications** | 2 |  |
| A new application for people with substance use | 2 | [36,43] |
